# Faster Release of Lumen-Loaded Drugs than Matrix-Loaded Equivalent in Polylactic Acid/Halloysite Nanotubes

**DOI:** 10.3390/ma12111830

**Published:** 2019-06-05

**Authors:** Chaitra Venkatesh, Oran Clear, Ian Major, John G. Lyons, Declan M. Devine

**Affiliations:** 1 Material Research Institute, Athlone Institute of Technology, Athlone N37 FK59, Ireland; c.venkatesh@research.ait.ie (C.V.); imajor@ait.ie (I.M.); 2 Faculty of Engineering and Informatics, Athlone Institute of Technology, Athlone N37 FK59, Ireland; A00251270@student.ait.ie (O.C.); slyons@ait.ie (J.G.L.)

**Keywords:** Aspirin, polylactic acid, halloysite nanotubes, nanocomposites, active pharmaceutical ingredients, melt extrusion, drug loading, drug delivery

## Abstract

Nanocomposite-based drug delivery systems with intrinsic controlled release properties are of great interest in biomedical applications. We report a novel polylactic acid (PLA)/halloysite nanotube (HNT) nanocomposite-based drug delivery system. PLA/HNT nanocomposites have shown immense potential for use in biomedical applications due to their favorable cyto- and hemo-compatibility. The objective of this study was to evaluate the release of active pharmaceutical ingredients (API) from PLA/HNT composites matrix and the effect of preloading the API into the lumen of the HNT on its release profile. Aspirin was used in this study as a model drug as it is a common nonsteroidal anti-inflammatory and antiplatelet agent widely used for various medical conditions. These two types of drug-loaded PLA/HNT nanocomposites were characterised by scanning electron microscopy (SEM), differential scanning calorimetry (DSC), Fourier transform infrared spectroscopy (FT-IR), surface wettability and mechanical testing. Statistical analysis was conducted on numerical data. Drug entrapment and in vitro drug release studies were conducted using UV spectrophotometry. Results indicate that aspirin was successfully loaded into the lumen of HNT, which resulted in the sustained release of aspirin from the nanocomposites. Furthermore, the addition of HNT into the polymer matrix increased the mechanical properties, indicating its suitability as a drug-eluting reinforcing agent.

## 1. Introduction

Hot-melt extrusion (HME) is widely used in the manufacture drug delivery systems for the controlled release of pharmaceuticals, as the mixing process of the drug and the polymer is efficient at controlled temperatures and pressures. This process can be used to melt or solubilize active pharmaceutical ingredients (APIs) in a polymer matrix to generate amorphous systems that effectively can improve solubility, modulate controlled release and have improved bioavailability [1,2,3,4,5,6].

Polylactic acid (PLA) is the most widely used biodegradable aliphatic polymer as it is obtained from renewable resources such as corn and rice starch [7,8]. PLA is approved by the FDA for direct contact with biological fluids as it is biocompatible and its degradation products are nontoxic [9,10]. PLA and PLA blends are most commonly used for medical applications such as stents, sutures, dermal fillers [11] and various drug delivery strategies such as nanosystems, films and fibrous matrices [12]. PLA has been studied as a drug release membrane, and the results have achieved sustained drug release for a long duration due to diffusion of the API from the metrics and bulk degradation [13].

PLA has been used to prepare biodegradable polymeric nanoparticles to improve their bioavailability, solubility and sustained release of APIs. In such cases, the APIs are confined inside the polymer membrane or uniformly dispersed on the surface of the polymer [14,15]. However, PLA on its own does not have all the mechanical properties required. The addition of nanoclays, specifically halloysite nanotubes (HNTs), has shown to improve the properties such as tensile strength, storage modulus, flexural properties and Young’s Modulus [16,17,18,19,20].

HNTs are a naturally occurring clay mineral which has been widely used in drug delivery systems as they are known to have a high aspect ratio, high surface area, nontoxicity, processability, fine particle size and biocompatibility [21]. The HNTs can be loaded with drugs due to its large outer surface and inner lumen [22]. Recent research has demonstrated the feasibility of loading of different drugs into the HNTs [23,24,25,26,27].

HNTs have also been studied for drug delivery in various polymer composites. Patel and researchers demonstrated the successful loading and sustained release of various drugs including antibiotics, antiseptics and disinfectants on electrospun HNT/PCL nanocomposites [27]. Qi and researchers successfully studied the double drug loading on HNTs and electrospinning with Poly (lactic-*co-*glycolic acid) (PLGA) [28]. To our knowledge, this is the first report for loading the HNTs with API and compounding with PLA by hot-melt extrusion (HME). We can hypothesise that the loading of the APIs into HNTs has better drug encapsulation and sustained drug release than when the APIs are compounded together with PLA and HNT. This drug-loaded nanocomposite can be employed for various medical applications requiring drug delivery such as scaffolds.

Aspirin (ASP) is a well-known pharmaceutical product used for various medical treatments. ASP is known to minimise inflammation, reduce body temperature and decrease the process of adhesion of platelets by blocking the production of hormones that participate in the process of inflammation, increasing body temperature and platelet fusion [29]. Due to the poor oral availability [30] and gastrointestinal side effects [31] of long-term oral administration of the aspirin, parenteral administration is required [32,33]. Also, ASP is an acidic drug which increases the water absorption of the polymer matrix, thereby affecting the rate of drug release and polymer degradation [32].

A number of studies have characterised drug loading and release profiles of ASP from polymer matrices. Devine et al. studied the encapsulation of aspirin in hydrophilic polymer coatings and copolymer hydrogels along with its associated release characteristics [34,35]. Shi et al. studied the loading of aspirin in different molecular weights of chitosan to analyse the release kinetics [36] and Zhang et al. recently demonstrated sustained drug release from aspirin-loaded chitosan nanoparticles [33].

In the current study, ASP was used as a model drug to study the effect of loading the API into the lumen of the HNTs prior to forming an HNT/PLA nanocomposite. This was compared to directly loading the API into the polymer matrix during compounding of the PLA/HNT nanocomposite. In previous work, the HME processing parameters were optimised in terms of enhanced mechanical properties of the final nanocomposite. In the current study, these optimised production settings were utilised to examine drug loading into the PLA/HNT nanocomposite structure to investigate the controlled release of API from these structures. We hypothesise that the loading of the ASP into the lumen of HNTs will result in enhanced sustained API release compared to direct loading of the API into the matrix. This drug-loaded nanocomposite may be employed for various medical applications requiring sustained drug delivery such as polymeric stents.

## 2. Experimental Details

### 2.1. Materials

PLA was obtained from Corbion, PLA LX 175 (Total Corbian, Gorinchem, The Netherlands). The density of the PLA was 1.24 g/cm^3^. HNT was obtained from Applied Minerals, DRAGONITE-HP (APPLIED MINERALS INC, Brooklyn. NY, USA) and had a density of 2.56 g/cm^3^. Acetyl salicylic acid (aspirin) was obtained from Sigma-Aldrich Ireland Ltd. (Wicklow, Ireland). The PLA and HNT were dried for 4 h in the oven at 80 °C. The moisture content of the PLA was measured before processing and 100 ppm moisture was utilised as an upper process limit.

### 2.2. Methods

#### 2.2.1. Drug Loading of HNTs

Aspirin was encapsulated into the lumen of the HNTs in two batches at ratios of 1:1 and 2:1. For the preparation of 1:1 ratio, 12 g of ASP were dissolved in 120 mL of ethanol under continuous stirring. Once the solution became transparent, 12 g of HNTs were added and stirred for 4 h at 700 rpm. The solution was then centrifuged to separate the solid part and dried overnight at 40 °C. Similarly, the 2:1 ratio was prepared using 12 g of HNTs and 6 g of ASP.

#### 2.2.2. Preparation of PLA/HNT/ASP Nanocomposites

**Figure 1:**
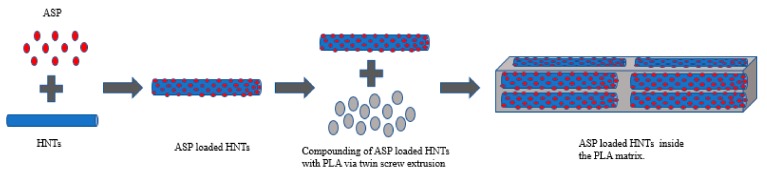
Schematic illustration PLA/(HNTs/ASP) nanocomposite by lumen-loading method for batch B1 and B5.

**Figure 2:**
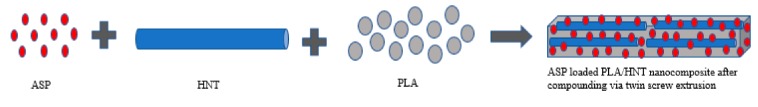
Schematic illustration of PLA/HNTs/ASP nanocomposite by matrix-loading method for batch B2 and B3.

Extrusion was performed by using APV (Model MP19TC (25:1), APV Baker, Newcastle-under-Lyme, UK) twin-screw compounder with 16 mm diameter screws. The temperature profile was maintained at (from die to the feeder) 140/135/130/125/120/115/110/50 °C. During extrusion, the screw speed was set at 140 rpm based on previous work. The mass fractions of PLA: HNTs were 100:0 and 95:5 for both lumen-loaded and matrix-loaded samples. The lumen-loaded HNT/ASP for both 1:1 and 2:1 ratios was tumble-mixed with PLA and extruded. These samples are referred to here as lumen-loaded. As a control, HNTs, ASP and the PLA were tumble-mixed and extruded at the same ratios. These samples are referred to here as matrix-loaded. The methods are illustrated in Figure 1 and Figure 2.

The extruded film was drawn through the three-roll calendar to form a continuous film. Finally, the films were punched into ASTM standard tensile test specimens for testing. The nomenclature are described below in Table 1.

#### 2.2.3. Characterisation of the Drug-Loaded Nanocomposite

##### Differential Scanning Calorimetry (DSC)

DSC analysis was carried out in triplicate using a DSC 2920 Modulated DSC (TA Instruments, New Castle, DE, USA). The nitrogen flow rate was maintained at 20 mL/min to prevent oxidation. Using indium as standard, the instrument was calibrated. Tests were conducted on samples weighing between 8 and 12 mg, while a sample weight of 2.0 ± 0.5 mg was used for ASP due to the bulk volume of the drug. Samples were measured on a Sartorius scale (MC 210 P, Sartorius Lab Instruments GmbH & Co. KG, Goettingen, Germany), which could read up to five decimal places. Samples were placed into non-perforated aluminum pans with an empty crimped aluminum pan used as a reference.

To remove the thermal history, the samples were heated from 20 °C to 220 °C at the rate of 30 °C/min where they were held isothermally for 10 min. Later, they were cooled from 220 °C to 20 °C at 30 °C/min. To record the thermal properties the samples were heated from 20 °C to 220 °C at the rate of 10 °C/min. Thus glass transition temperature, cold crystallisation temperature and melting temperature of each sample were recorded.

##### Mechanical Testing

Mechanical properties of the PLA/HNTs composites were characterised by tensile testing, using Lloyd Lr10k tensometer (Ametek ltd, West Sussex, UK). A 2.5 kN load cell was used on ASTM standard test specimens. The strain rate was maintained at 50 mm/min (n = 12). Data was recorded using NexygenTM software (Ametek ltd). The tensile tests were carried out in adherence to ASTM D 882. Twelve test specimens were analysed per group. Dimensional measurements were completed on each sample prior to testing. For each tested sample Young’s Modulus, stiffness, percentage strain at maximum load and stress at maximum load were recorded

##### Fourier Transform Infrared (FTIR) Spectroscopy

FTIR was carried out on a Perkin Elmer Spectrum One fitted with a universal ATR sampling accessory (Perkin Elmer, Waltham, MA, USA). The recordings of all the data were in the spectral range of 4000–650 cm^−1^ at 21 °C. A resolution of 1 cm^−1^ was employed, a fixed universal compression force of 70–80 N with four scans per sample was recorded. Spectrum software was used for subsequent analysis.

##### Goniometry (Surface Wettability)

The assessment of surface wettability of the composites was done using First Ten Angstroms FTA32 goniometer (FTA Europe, Cambridge, UK). In this test, the Sessile Drop contact angle technique was utilised using distilled water as the probe liquid. Five measurements at various places on the films were taken for each composite sample.

##### Evaluation of Drug-Loaded Efficiency

Aspirin encapsulation and loading efficiency were studied by separating the ASP-loaded HNTs from the free drug present in the aqueous medium by method of centrifugation. Centrifugation was carried out at room temperature for 30 min at 700 rpm. A UV spectrophotometer was used to determine the amount of free aspirin at 276 nm. A standard calibration curve of concentration versus absorbance was plotted. The ASP encapsulation efficiency (EE) and the ASP loading capacity (LC) of the process were calculated from the equations below [28,36,37]

(1) $$EE=\frac{\left(total\;amount\;of\;ASP-free\;ASP\right)}{total\;amount\;of\;ASP}\times 100$$

(2) $$LC=\frac{total\;amount\;of\;ASP-free\;ASP}{nanoparticles\;weight}\times 100$$

##### In Vitro Drug Release Properties

The drug release mechanism was studied by placing the samples in simulated body fluid (SBF) pH 7.4 in DISTEK 2100 dissolution apparatus (Distek, Inc. North Brunswick, NJ, USA). Each vessel of the dissolution apparatus contained 500 mL of SBF at 36.7 °C with rotation speed of the stirring element shaft at 50 rpm for three weeks. At selected time intervals, 5 mL of the release buffer was removed for UV analysis and replaced with an equal volume of fresh buffer. The amount of released aspirin was measured by UV Spectrophotometer (Shimadzu Europa GmbH, Duisburg, Germany) at 298 nm. This experiment was carried out in triplicate to obtain the release profile of the ASP in relation to time.

##### Statistical Analysis

Statistical analysis of the tensile test results, DSC measurements and the surface wettability measurements were carried out by analysis of variance (one-way ANOVA) with a Tukey Post hoc test for multiple comparisons using Minitab 17 Statistical Software (Minitab Ltd., Coventry, UK). All the values were considered at a 95% confidence interval, and differences were considered significant when *p* ≤ 0.05.

## 3. Results

### 3.1. Preparation of Drug-Loaded HNTs

**Figure 3:**
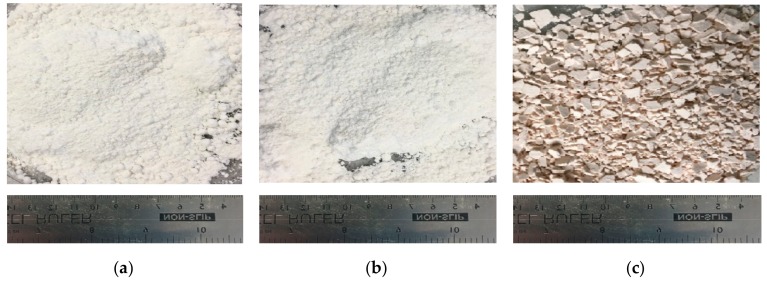
Photograph of (**a**) ASP, (**b**) HNTs, (**c**) ASP-loaded HNTs after drying in the oven at 40 °C overnight. The resultant blend had a pale pink colour as compared to the white colour of the ASP and HNTs individually.

Aspirin loading into HNTs was performed without any difficulty. Prior to loading, the ASP and HNTs were received in white powder form. The ASP-loaded HNTs underwent a subtle colour change to a pale (mild pink-colored) powder as seen in Figure 3.

The ASP encapsulation efficiency (EE) for 2:1 and 1:1 ratios was calculated to be 45.3% and 66.6%, respectively. The ASP loading capacity (LC) for 2:1 and 1:1 ratios was 22.6% and 66.6%, respectively.

### 3.2. Processing of PLA and HNT through Melt Extrusion

**Figure 4:**
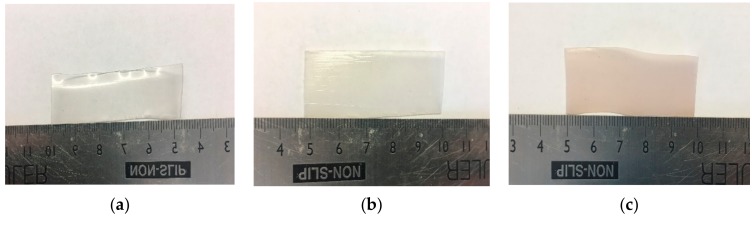
Photographs showing (**a**) virgin PLA films are transparent, (**b**) PLA/HNT nanocomposite films are opaque, (**c**) ASP-loaded PLA/HNT nanocomposite are mild pink in colour.

Virgin PLA, PLA/HNTs, PLA/(HNT-ASP) and PLA/HNT/ASP nanocomposites were melt-compounded followed by calendaring at screw speeds of 140 rpm without difficulty. The extruded PLA remained transparent. However, the PLA/HNTs nanocomposites changed in colour from transparent to opaque. The PLA/(HNT-ASP) and PLA/HNT/ASP nanocomposites were opaque with mild pink colour as shown in Figure 4.

### 3.3. Differential Scanning Calorimetry (DSC)

**Figure 5:**
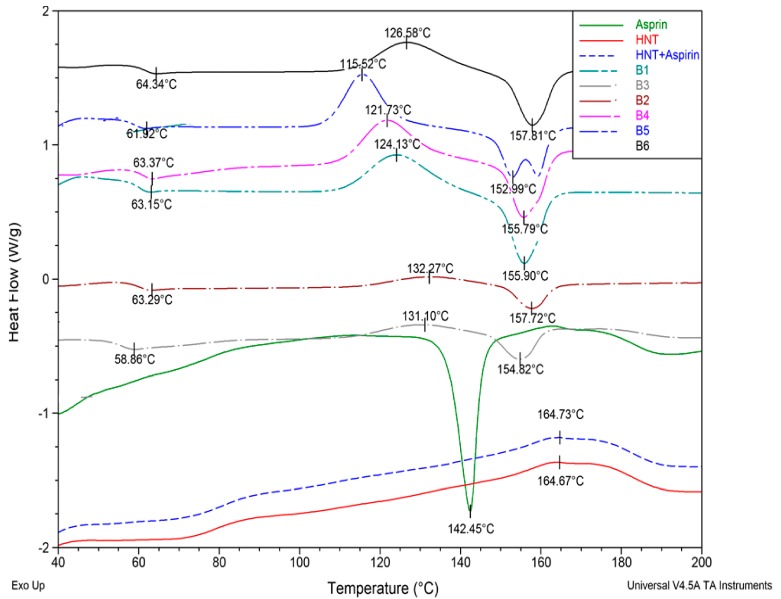
Thermograms of B1, B2, B3, B4, B5, B6, HNT, ASP and HNT+ASP. There is no change in T_g_ due to ASP loading when compared to the virgin PLA and PLA/HNT nanocomposite.

The thermal properties of the nanocomposites following drug encapsulation were analysed using DSC. Figure 5 shows the thermograms of all the batches including the ASP and HNTs for comparison. Aspirin shows its characteristic melting peak at 142.45 °C as expected. However, this peak is not evident in the ASP-loaded HNTs curve.

From analysis of the thermograms of the nanocomposites, the results indicate that the glass transition temperature (T_g_) does not show any significant change due to the addition of ASP in the nanocomposite. However, the cold crystallisation temperature exhibits a significant decrease when the ASP is lumen-loaded at a 2:1 ratio when compared to other nanocomposite batches. It is interesting to note that the cold crystallization temperature increases and there is little or no curve for the cold crystallization observable in the matrix-loaded batches B2 and B3 of both 1:1 and 2:1 ratios. The melting temperature of the PLA (B6) is 157 °C, which decreases only for the lumen-loaded 2:1 nanocomposite (B5) and there is no significant difference in the melting temperature for other samples.

### 3.4. Mechanical Testing

**Figure 6:**
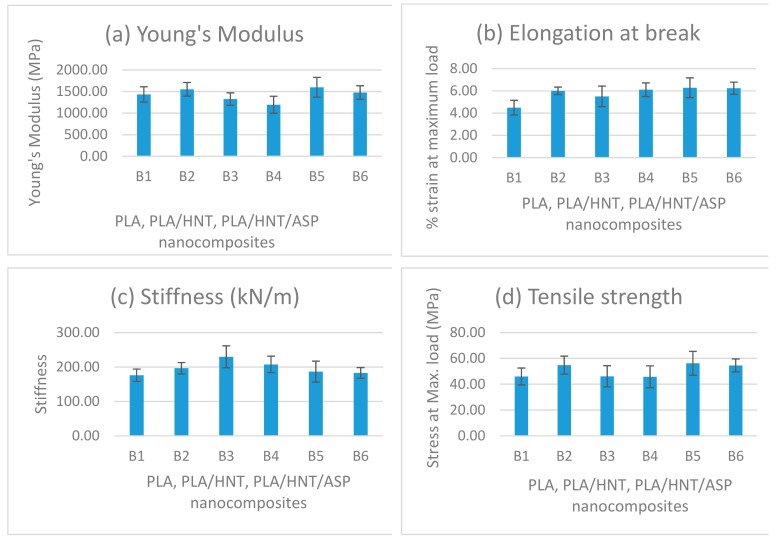
Mechanical properties of the nanocomposites are shown in the above graph. (**a**) Young’s modulus of the preloaded 2:1 nanocomposite (B5) is significantly higher with *p-*value of 0.001. (**b**) Elongation at break does not show any significant change. (**c**) Stiffness significantly increases for 2:1 matrix-loaded nanocomposite (B3) with a *p-*value of 0.001. (**d**) Tensile strength significantly increases for preloaded 2:1 nanocomposite (B5) batch with *p*-value of 0.001.

Mechanical testing was conducted to analyse and compare the effect of the drug on the mechanical properties of the nanocomposite. The analysis of the mechanical properties of all the batches produced is depicted in Figure 6.

In comparison to the virgin PLA (B6) and PLA/HNT nanocomposite (B4), Young’s modulus and the tensile strength significantly increased for the preloaded 2:1 nanocomposite (B5), whereas stiffness significantly increased for matrix-loaded 2:1 nanocomposite (B3), with no significant change in the elongation at break for any batch. It is interesting to note that 2:1 ratio of ASP loading changed the mechanical properties of the nanocomposite when compared to the 1:1 loading of the ASP.

### 3.5. Fourier Transfer Infrared Spectroscopy (FTIR)

The chemical structure of PLA is (C_3_H_4_O_2_)_n_, HNT is Al_2_Si_2_O_5_·(OH)_4.2_(H_2_O) and ASP is C_9_H_8_O_4_. FTIR analysis was performed to study the chemical reaction in PLA/HNTs composites. Virgin PLA exhibited characteristic peaks at 3571 cm^−1^ corresponding to –OH stretch, peaks at 2995 cm^−1^ and 2946.34 cm^−1^ were attributed to CH stretch, and the peaks at 1750 cm^−1^ indicated –C=O carbonyl group [38]. The spectrum of HNTs displayed peaks at 3693.26 cm^−1^ and 3623.39 cm^−1^ which can be assigned to O–H group and peak at 906.81 cm^−1^ to Al–OH group.

The spectra of PLA/HNTs composites displayed characteristic peaks of both PLA and HNTs. The functional groups of HNT such as O–H stretching of the hydroxyl group are displayed by the characteristic peak located at 3696.39 cm^−1^ and 3626.39 cm^−1^.

**Figure 7:**
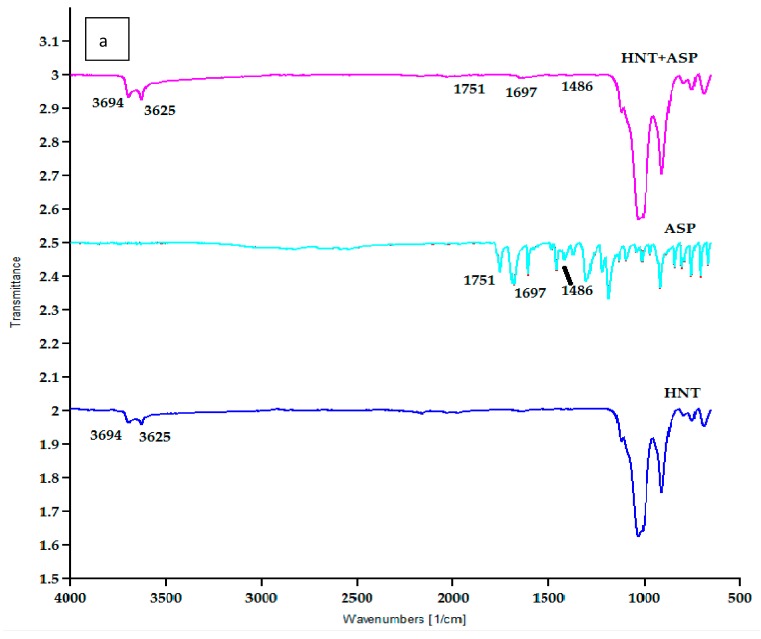

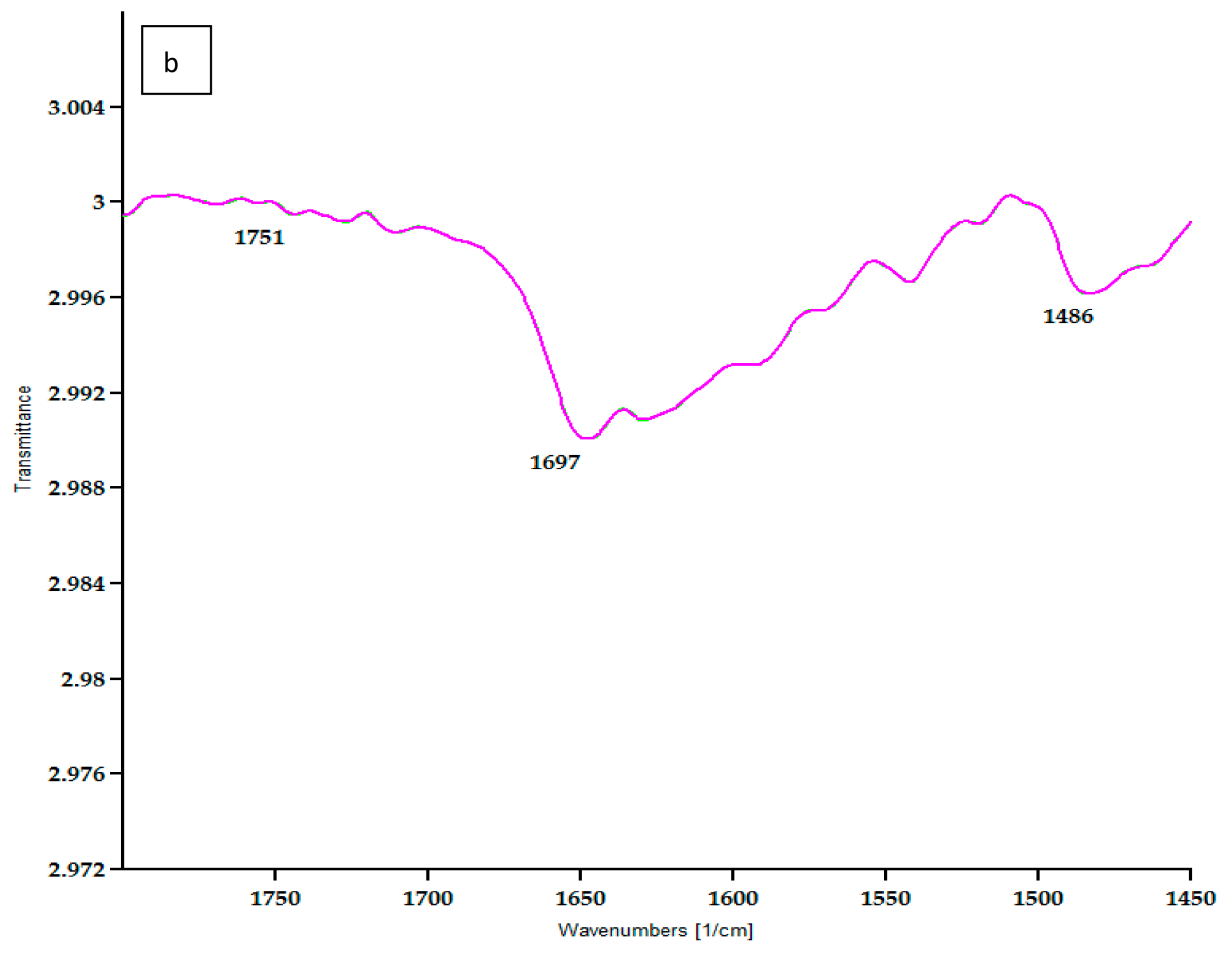
(**a**) FTIR spectra of HNT, ASP and ASP-loaded HNTs samples. The peaks of ASP at 1751 cm^−1^, 1697 cm^−1^ and 1486 cm^−1^ correspond with the peaks of ASP-loaded HNTs at 1751 cm^−1^, 1697 cm^−1^ and 1486 cm^−1^. This indicates the drug is loaded in the HNT. (**b**) Higher magnification of HNT + ASP spectrum.

The analysis of the spectra of HNTs, ASP and the ASP lumen-loaded HNTs is shown in Figure 7. The spectra indicate the characteristic peaks of ASP at 1751 cm^−1^ corresponding to C=O stretching, 1697 cm^−1^ corresponding to –COO stretch and 1486 cm^−1^ from antisymmetric stretching vibration of a carboxylate form of aspirin [26,39]. These peaks were observed in the lumen-loaded HNT at 1751 cm^−1^, 1697 cm^−1^ and 1486 cm^−1^. This indicates the ASP molecules had been attached on the HNTs after drug loading.

### 3.6. Surface Wettability

**Figure 8:**
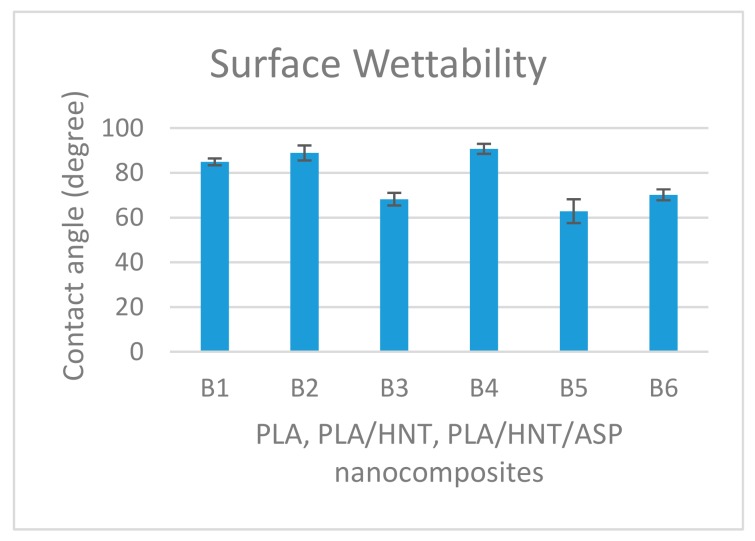
Graphical representation of contact angle measurements for all batches indicates the hydrophilicity nature of B5 and B3 batch of 2:1 ratio of both preloading and matrix-loading which is comparable to native PLA which has a contact angle of 70°. The B1 and B2 samples with a 1:1 ratio tend to be hydrophobic, exhibiting a contact angle of ca. 90°.

As showen in Figure 8, the contact angle of the B5 and B3 significantly decreases when compared to B1 and B2 (with *p* ≤ 0.04, for all comparisons), whereas the contact angle measurement significantly increases for B1 and B2 when compared to B6 with *p* ≤ 0.035 (for both comparisons).

### 3.7. Drug Release

**Figure 9:**
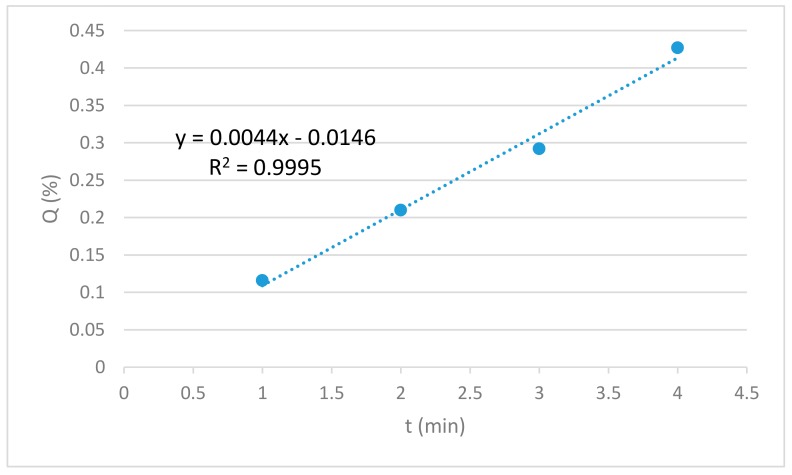
Standard curve of API release for the lowest to the highest concentration of API.

The amount of API released from the lowest to the highest concentration is plotted against the time of release. The graphical representation of the amount of API release (Q%) versus time (t) shows linearity as seen in Figure 9. Thus, the drug release rate is independent of the concentration of the API. In this study, the release kinetics of aspirin showed the best fit for zero-order model (R^2^ = 0.9995) which represents slow release of drug and is expressed by the equation Q_t_ = Q_0_ + K_0_ t where Q_t_ is the amount of drug dissolved at time t, Q_0_ is the initial amount of drug in the solution and K_0_ is the zero-order release constant expressed in units of concentration/time. The data obtained from in vitro drug release studies were plotted as cumulative amount of drug released versus time.

**Figure 10:**
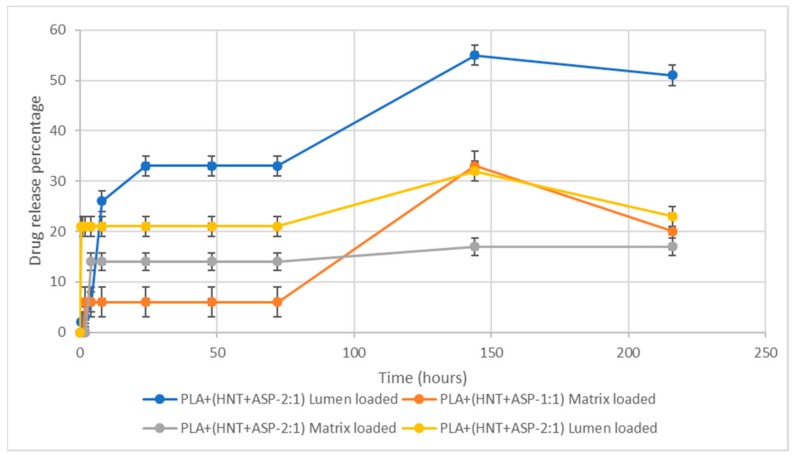
In vitro release kinetics show a general burst release of API followed by no further release until at least 72 h. The lumen samples exhibited a higher release rate compared to the matrix-loaded counterparts.

The API release profiles for all batches tested are presented in Figure 10. Batches B1 and B2 had an API loading ratio of 1:1, HNT:API. From API release studies, it was found that lumen-loaded B1 samples of 1:1 ratio exhibited a burst release of 26% in 8 h. This was followed by a slow release of 33% up to 72 h and 55% at 144 h. On the ninth day, there was a gradual decrease to 51% release. Conversely, the release from the matrix-loaded B2 was recorded at 6% after 2 h, with no further release observed until the sixth day when 33% release was detected, which was reduced to 20% release on the ninth day.

Batches B3 and B5 had an API loading ratio of 2:1, HNT:API. The matrix-loaded B3 samples exhibited a burst release of 14% after 4 h, which again did not increase until the ninth day when 17% API release was detected. The lumen-loaded B5 samples exhibited a burst release of 21% within 1 h with no further API release detected until after six days when 32% API release was recorded, which decreased to a release of 23% on the ninth day.

## 4. Discussion

The present study explored the feasibility of controlled release of API from PLA/HNT composites. It also compared the release of API preloaded into the lumen of the HNT component of the nanocomposite compared to API loaded directly into the matrix of the nanocomposite.

The polymer matrix utilized in this study was PLA. This polymer was selected as it is a commonly used biomedical polymer [9] which has a glass transition temperature of 60 °C. This is important as the polymer selected for the HME must have deformation potential and the glass transition temperature must be less than the melting point of the drug, which in the case of aspirin is 135 °C [1]. Aspirin was selected to molecularly disperse inside the polymer matrix by the generation of pressure during HME and melting the various components within the mixture (drug, polymer and nanoparticle) at a set temperature [1].

HNTs were selected as they have shown great potential as a reinforcing agent in a PLA nanocomposite system [40], and structurally, the large surface area and the inner lumen of HNTs make it suitable for the drug loading and sustained release. HNTs have been shown to increase drug effectiveness without increasing the concentration of the drug as it is slowly released from the HNTs [22].

### 4.1. Drug Loading

The HNTs are normally mixed with a saturated solution of a chosen API in ethanol, water or other solvents to entrap hydrophilic drugs [41]. However, the low dissolution rate of the ASP in water reduces the drug absorption rate and also toughens the maintenance of its concentration inside the body [26]. Hence, in this study, the ASP was dissolved in ethanol and HNTs were added to the solution for the drug loading.

The API loading on the HNT is either the API is encapsulated in the inner lumen, or it may be bound to the outer surface. The negatively charged outer surface of the HNT could encapsulate the ASP through hydrogen bonding between the carbonyl group of the ASP and the hydroxyl or the siloxane group of the HNT. When the vacuum is applied during the API loading, the positively charged inner lumen of the HNT could entrap the ASP [26,41]. Most likely, the ASP is distributed in both inner and outer lumen which could lead to initial burst release from the outer surface and gradual and slow release of the API from the inner lumen [22].

The ASP encapsulation efficiency (EE) and the loading capacity (LC) were better for 1:1 loading ratio. Similar results were reported by Qi et al. in their API release study from electrospun PLGA/HNT nanofiber composites. In this work, it was hypothesised that the fast deposition and aggregation of HNTs at higher concentrations lead to relatively less surface area being exposed to the API, which resulted in lower EE and LCs [28].

### 4.2. Melt Extrusion

Twin-screw extrusion is a high-shear process and it has been shown that increases in screw speed during compounding increase melt shear and aid HNT distribution in the PLA matrix. As such, a screw speed of 140 rpm was utilised in this study based on previous findings [42].

The temperature profile set for melting the PLA during extrusion was between 140 and 200 °C. The temperature profiles for the extrusion process should be 20–40 °C above the T_g_ of the polymer [43]. However, since the melting temperature of the ASP is 135 °C, the extrusion was performed above 140 °C. This enabled ASP to disperse molecularly inside the polymer matrix [1].

### 4.3. Thermal Characteristics

The characteristic melting peak of ASP did not appear in the DSC thermograms of ASP-loaded HNTs. This could indicate the loading of the ASP inside the cavity of the HNTs [44]. This could also be indicative of the amorphous state of the drug [45].

The glass transition temperature is the measure of rigidity [46]. The addition of HNTs or the ASP has a minimal effect on the rigidity of the PLA backbone chain. The cold crystallisation temperature exhibited a significant decrease when the ASP was preloaded into the lumen of the HNT at a 2:1 ratio when compared to other batches of the nanocomposites. A reduction in Tcc of PLA/ASP-HNTs composites has been reported to be indicative of heterogeneous nucleation, where thinner or less perfect crystalline lamella are formed [8,47]. This is further supported by the twin melting peak of the nanocomposite, which is indicative of the melting of imperfect crystalline segments.

Conversely, the matrix-loaded nanocomposites showed a negligible or nearly no cold crystallisation curve for both 2:1 and 1:1 ratios. In a previous study by Shi et al., the addition of plasticizer into the PLA composites has been shown to result in very small or no cold crystallisation peak indicating the increased mobility of PLA chains and advanced crystallisation of the nucleating agents prior to cooling [48]. Hence, a negligible or nearly no cold crystallisation curve for both 2:1 and 1:1 ratio ASP-loaded nanocomposite may be due to the plasticizing effect of the ASP when processed together with PLA and HNT.

### 4.4. Mechanical Properties

The mechanical properties of the nanocomposites varied with HNT:ASP loading and encapsulation method. The coaxial alignment of HNTs in the PLA matrix enables the efficient load transfer from PLA to HNTs, which improves the mechanical properties.

Results from the current study indicate that the 2:1 lumen-loaded nanocomposite (B5) exhibited an increase in Young’s Modulus and the tensile strength compared to the PLA/HNT. This is likely related to the plasticising effect of ASP on the system as observed in DSC data which allowed molecular chains to slide over each other more easily under tension. It is interesting to note the drug inclusion in the nanocomposite does not deteriorate the strength, unlike the study conducted by Patel and researchers where the tensile strength was reduced when the drug-loaded HNTs were loaded into the PLA matrix [27]. However, in their study, the method of processing was electrospinning. Here, the process of extrusion would have induced HNT dispersion through melt shearing [42]. Similarly, there was a significant increase in stiffness for matrix-loaded 2:1 nanocomposite (B3) when compared to the other batches.

When the drug-loaded nanocomposites are compared to the PLA/HNT nanocomposites (B4), the observed increases in mechanical properties could be due to the interactions of the ASP molecules with the HNTs, which enhance their interfacial adhesion and dispersion within the matrix [48].

### 4.5. Fourier Transform Infrared (FTIR) Spectroscopy

The spectrum of ASP-loaded HNTs shows similarities to the HNT spectrum. However, the characteristic peaks of ASP are evident at low intensities as indicated by peaks at 1751 cm^−1^, 1486 cm^−1^ and 1697 cm^−1^ which are characteristic of C=O stretch, –COO stretch and antisymmetric stretching vibration of a carboxylate form of aspirin in ASP. Similar analyses and findings were reported by Lun and researchers [26]. As the peaks of ASP are not intense and very similar to HNT peaks, this could indicate the loading of the ASP in the HNT cavity, which is relatable to the thermal analysis of the same. This also could indicate the surface of HNTs are free of residual ASP [44].

The spectra of all the drug-loaded PLA/HNT nanocomposite films were very similar to the PLA spectrum with no peaks of ASP observed. This could be due to the insensitivity of the FTIR technique or factually, the vibration bands of ASP or HNTs could be overlapping with PLA bands [28].

### 4.6. Surface Wettability

PLA exhibited a hydrophilic nature, whereas HNTs were relatively hydrophobic in agreement with previous work [20,49]. ASP is a hydrophilic drug [32]. The hydrophilicity of B5 and B3 indicate that the loading ratio of 2:1 of HNTs:ASP irrespective of the type of processing decreases the contact angle of the nanocomposites. This could be attributed to the higher agglomeration of HNT which resulted in a lower drug uptake. This could result in a higher amount of ASP in the matrix system which had a plasticising effect and rendered the composite more hydrophilic compared to the 1:1 samples. Similar findings were seen by Patel and researchers in their study with PCL/HNT nanocomposites with antibacterial drugs [27].

### 4.7. Drug Release

**Figure 11:**
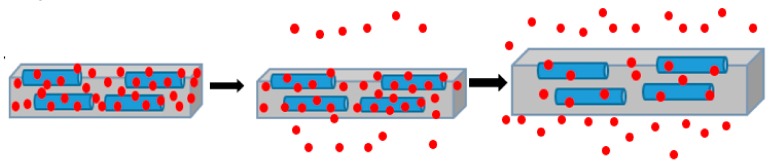
Schematic representation of API release mechanism for PLA/HNT/ASP nanocomposite through diffusion and swelling.

ASP release from the nanocomposite systems indicate that both the HNT:ASP ratio and entrapment method had a large effect on ASP release. The API release mechanism for the PLA/HNT/ASP nanocomposite was through diffusion and swelling of the nanocomposite as shown in Figure 11.

From ASP data, it was shown that the matrix-loaded samples did not have any burst release and that the release began following 8 h incubation, which would indicate that the drug had started to diffuse through the hydrophilic PLA matrix. Conversely, the lumen-loaded samples did have a burst release of 21–28% in the first 1–8 h. This may be attributed to the ASP washing from the outer lumen of the HNT, which were close to the surface of the PLA. In terms of HNT:ASP ratio, and in agreement with drug-loading findings, the 1:1 samples yielded a higher total API release at the end of the nine-day experiment. From the literature, it has been reported that the reduction in initial burst release may be due to the effect of the plasticizer in the polymer matrix [32]. The slow drug release suggests the presence of major amounts of ASP in the lumen of the HNTs at that time point [28]. The thermal properties of test samples in this study show that the matrix-loaded ASP for both 2:1 and 1:1 ratio nanocomposite had negligible cold crystallisation curves, which could be due to the plasticizing effect of the ASP when loaded directly into the PLA matrix. Plasticizers, which are capable of increasing flexibility of the polymers and can reduce the degradation of the thermolabile drugs [4]. The higher percentage of drug release after 140 h can be attributed to the gradual degradation of the PLA which had extended the retention time of the API [15].

It is interesting to note the reduction in the percentage release of the API from the nanocomposites on the ninth day except for the 1:1 matrix-loaded nanocomposite (B3). This could be due to the presence of the ASP in the inner lumen of the HNT, which gradually diffused to the PLA matrix during the nine days. Thus the loading of the ASP onto the HNT potentially on the inner lumen reduces the burst release and aids sustained release over a period of time. In this study, the matrix-loaded samples did not have a burst release and were followed by a sustained release, which could be due to the better dispersion and loading of the ASP on the HNT during extrusion.

## 5. Conclusions

The drug–polymer nanocomposite delivery system of ASP-PLA/HNT nanocomposite exhibits a sustained release performance. The encapsulation efficiency and the loading capacity were better for 1:1 ratio loading of HNT:ASP. FTIR characterisation revealed the presence of ASP in the ASP-loaded HNTs. DSC data demonstrated the API–polymer interaction between aspirin and PLA/HNTs. The API–polymer interaction affected the release of aspirin, resulting in a sustained release action. Lumen-loaded samples had burst release and overall higher release of the API when compared to the matrix-loaded samples, which did not have initial burst release. In terms of ratios, 1:1 for HNT:ASP resulted in a higher total of API released at the end of the nine-day experiment. Hence, we have shown that the release of API can be controlled using different nanocomposite compositions.

## Figures and Tables

**Table 1:** Description of the prepared sample batches, the method of processing, composition and ratio of each component for each batch.

Batch	Type of Loading	Composition	HNTs:ASP Ratio
B1	Lumen-loaded	95% PLA, 5% (HNT+ASP)	1:1
B5	Lumen-loaded	95% PLA, 5% (HNT+ASP)	2:1
B2	Matrix-loaded	95% PLA, 5% (HNT+ASP)	1:1
B3	Matrix-loaded	95% PLA, 5% (HNT+ASP)	2:1
B4	Matrix-loaded	95% PLA, 5% HNT	-
B6	Matrix-loaded	100%PLA	-

## Nomenclature

HME	Hot-melt extrusion
API	Active pharmaceutical ingredients
PLA	Polylactic acid
HNTs	Halloysite nanotubes
ASP	Aspirin (acetyl salicylic acid)
Lumen loading	ASP loading on HNTs and processing with PLA
Matrix loading	ASP, HNTs and PLA are mixed and processed
B1	1:1 ratio of lumen loading
B2	1:1 ratio of matrix loading
B3	2:1 ratio of matrix loading
B4	Processed PLA/HNT nanocomposite (matrix loading)
B5	2:1 ratio of lumen loading
B6	Processed virgin PLA (matrix loading)
DSC	Differential scanning calorimetry
T_g_	Glass transition temperature
T_cc_	Cold crystallisation temperature
T_m_	Melting temperature
FTIR	Fourier transfer infrared spectroscopy
SEM	Scanning electron microscopy

## References

[1] Fule R., Paithankar V., Amin P. (2016). Hot melt extrusion based solid solution approach: Exploring polymer comparison, physicochemical characterization and in-vivo evaluation. Inter. J. Pharm..

[2] Lyons J.G., Holehonnur H., Devine D.M., Kennedy J.E., Geever L.M., Blackie P., Higginbotham C.L. (2007). The incorporation of an organically modified layered silicate in monolithic polymeric matrices produced using hot melt extrusion. Mater. Chem. Phys..

[3] Martinez-Marcos L., Lamprou D.A., McBurney R.T., Halbert G.W. (2016). A novel hot-melt extrusion formulation of albendazole for increasing dissolution properties. Inter. J. Pharm..

[4] Patil H., Tiwari R.V., Repka M.A. (2016). Hot-Melt Extrusion: From Theory to Application in Pharmaceutical Formulation. AAPS PharmSciTech.

[5] Maniruzzaman M., Boateng J.S., Snowden M.J., Douroumis D. (2012). A review of hot-melt extrusion: Process technology to pharmaceutical products. ISRN Pharm..

[6] Saerens L., Vervaet C., Remon J.P., De Beer T. (2014). Process monitoring and visualization solutions for hot-melt extrusion: A review. J. Pharm. Pharmacol..

[7] Healy A.V., Waldron C., Geever L.M., Devine D.M., Lyons J.G. (2018). Degradable Nanocomposites for Fused Filament Fabrication Applications. J. Manuf. Mater. Process..

[8] Chen Y., Murphy A., Scholz D., Geever L.M., Lyons J.G., Devine D.M. (2018a). Surface-modified halloysite nanotubes reinforced poly(lactic acid) for use in biodegradable coronary stents. J. Appl. Polym. Sci..

[9] Chen Y., Geever L.M., Killion J.A., Lyons J.G., Higginbotham C.L., Devine D.M. (2016). Review of Multifarious Applications of Poly (Lactic Acid). Polym. Plastics Technol. Eng..

[10] Farah S., Anderson D.G., Langer R. (2016). Physical and mechanical properties of PLA, and their functions in widespread applications — A comprehensive review. Adv. Drug Deliv. Rev..

[11] Tyler B., Gullotti D., Mangraviti A., Utsuki T., Brem H. (2016). Polylactic acid (PLA) controlled delivery carriers for biomedical applications. Adv. Drug Deliv. Rev..

[12] Saini P., Arora M., Kumar M.N.V.R. (2016). Poly(lactic acid) blends in biomedical applications. Adv. Drug Deliv. Rev..

[13] Mhlanga N., Ray S.S. (2015). Kinetic models for the release of the anticancer drug doxorubicin from biodegradable polylactide/metal oxide-based hybrids. Inter. J. Biol. Macromol..

[14] Nagavarma B.V.N., Yadav H.K.S., Ayaz A., Vasudha L.S., Shivakumar H.G. (2012). Different Techniques for preparation of polymeric nanoparticles—A review. Asian J. Pharm. Clinical Res..

[15] Sha L., Chen Z., Chen Z., Zhang A., Yang Z. (2016). Polylactic acid based nanocomposites: Promising safe and biodegradable materials in biomedical field. Inter. J. Polym. Sci..

[16] Laboratory of Polymeric and Composite Materials. http://morris.umh.ac.be/smpc/poster.aspx.

[17] Prashantha K., Lacrampe M.-F., Krawczak P. (2013). Halloysite Nanotubes-Polymer Nano composites:A New Class of Multifaceted Materials. Advan. Mater. Manuf. Charact..

[18] Wu W., Cao X., Zhang Y., He G. (2013). Polylactide/halloysite nanotube nanocomposites: Thermal, mechanical properties, and foam processing. J. Appl. Polym. Sci..

[19] Castro-aguirre E., Auras R., Selke S., Rubino M., Marsh T. (2018). Impact of Nanoclays on the Biodegradation of Poly (lactic acid) Nanocomposites. Polymers.

[20] Chen Y., Geever L.M., Killion J.A., Lyons J.G., Higginbotham C.L., Devine D.M. (2017). Halloysite nanotube reinforced polylactic acid composite. Polym. Compos..

[21] Gaaz T., Sulong A., Kadhum A., Al-Amiery A., Nassir M., Jaaz A. (2017). The Impact of Halloysite on the Thermo-Mechanical Properties of Polymer Composites. Molecules.

[22] Lvov Y.M., DeVilliers M.M., Fakhrullin R.F. (2016). The application of halloysite tubule nanoclay in drug delivery. Expert Opin. Drug Deliv..

[23] Abdullayev E., Lvov Y. (2011). Halloysite Clay Nanotubes for Controlled Release of Protective Agents. J. Nanosci. Nanotechnol..

[24] Fu L., Yang H., Tang A., Hu Y. (2017). Engineering a tubular mesoporous silica nanocontainer with well-preserved clay shell from natural halloysite. Nano Res..

[25] Leporatti S. (2017). Halloysite clay nanotubes as nano-bazookas for drug delivery. Polym. Inter..

[26] Lun H., Ouyang J., Yang H. (2014). Natural halloysite nanotubes modified as an aspirin carrier. RSC Adv..

[27] Patel S., Jammalamadaka U., Sun L., Tappa K., Mills D. (2015). Sustained Release of Antibacterial Agents from Doped Halloysite Nanotubes. Bioengineering.

[28] Qi R., Guo R., Shen M., Cao X., Zhang L., Xu J., Yu J., Shi X. (2010). Electrospun poly(lactic-co-glycolic acid)/halloysite nanotube composite nanofibers for drug encapsulation and sustained release. J. Mater. Chem..

[29] Katerina M. (2008). Aspirin–friend or foe?. THTY.

[30] Khadka P., Ro J., Kim H., Kim I., Kim J.T., Kim H., Cao J.M., Yun G., Lee J. (2014). Pharmaceutical particle technologies: An approach to improve drug solubility, dissolution and bioavailability. Asian J. Pharm. Sci..

[31] Ng J.C., Yeomans N.D. (2018). Infection and the risk of upper gastrointestinal bleeding in low dose aspirin users: Systematic review and meta-analysis. Med. J. Aust..

[32] Tang Y., Singh J. (2008). Controlled delivery of aspirin: Effect of aspirin on polymer degradation and in vitro release from PLGA based phase sensitive systems. Inter. J. Pharm..

[33] Zhang J., Ma S., Gene H. (2017). Guided bone regeneration with asymmetric collagen-chitosan membranes containing aspirin- loaded chitosan nanoparticles. Inter. J. Nanomed..

[34] Devine D.M., Geever L.M., Higginbotham C.L. (2005). Drug release from a N-vinylpyrrolidinone/acrylic acid lubricious hydrophilic coatin. J. Mater. Sci..

[35] Devine D.M., Devery S.M., Lyons J.G., Geever L.M., Kennedy J.E., Higginbotham C.L. (2006). Multifunctional polyvinylpyrrolidinone-polyacrylic acid copolymer hydrogels for biomedical applications. Inter. J. Pharm..

[36] Shi Y., Wan A., Shi Y., Zhang Y., Chen Y. (2014). Experimental and mathematical studies on the drug release properties of aspirin loaded chitosan nanoparticles. BioMed. Res. Inter..

[37] Das S., Banerjee R., Bellare J. (2005). Aspirin loaded albumin nanoparticles by coacervation: Implications in drug delivery. Trends Biomater. Artif. Org..

[38] Garlotta D. (2002). A Literature Review of Poly (Lactic Acid). J. Polym. Environ..

[39] Semalty A., Semalty M., Singh D., Rawat M.S.M. (2010). Development and Characterization of Aspirin-Phospholipid Complex for Improved Drug Delivery. J. Pharm. Sci..

[40] Višnjić D., Lalić H., Dembitz V., Banfić H. (2014). Metabolism and differentiation. Period. Biol..

[41] Qi R., Guo R., Zheng F., Liu H., Yu J., Shi X. (2013). Controlled release and antibacterial activity of antibiotic-loaded electrospun halloysite/poly(lactic-co-glycolic acid) composite nanofibers. Coll. Surf. B Biointerfaces.

[42] Venkatesh C., Chen Y., Cao Z., Brennan S., Major I., Lyons J., Devine D. Increased screw speed has positive effect on Polylactic Acid-Halloysite Nanotubes nanocomposite.

[43] Jani R., Patel D. (2014). Hot melt extrusion: An industrially feasible approach for casting orodispersible film. Asian J. Pharm. Sci..

[44] Gao M., Lu L., Wang X., Lin H., Zhou Q. (2017). Preparation of a novel breviscapine-loaded halloysite nanotubes complex for controlled release of breviscapine Preparation of a novel breviscapine-loaded halloysite nanotubes complex for controlled release of breviscapine. Mater. Sci. Eng..

[45] Balogh A., Domokos A., Farkas B., Farkas A., Rapi Z., Kiss D. (2018). Supporting Information Continuous End-to-End Production of Solid Drug Dosage Forms: Coupling Flow Synthesis and Formulation by Electrospinning. Chem. Eng. J..

[46] Devine D.M., Hoctor E., Hayes J.S., Sheehan E., Evans C.H. (2017). Extended release of proteins following encapsulation in hydroxyapatite/chitosan composite scaffolds for bone tissue engineering applications. Mater. Sci. Eng. C.

[47] Dong Y., Marshall J., Haroosh H.J., Mohammadzadehmoghadam S., Liu D., Qi X., Lau K.T. (2015). Polylactic acid (PLA)/halloysite nanotube (HNT) composite mats: Influence of HNT content and modification. Compos. Part A Appl. Sci. Manuf..

[48] Shi X., Zhang G., Phuong T.V., Lazzeri A. (2015). Synergistic effects of nucleating agents and plasticizers on the crystallization behavior of Poly(lactic acid). Molecules.

[49] Lazzara G., Massaro M., Milioto S., Riela S. (2017). Halloysite-based bionanocomposites. Handb. Compos. Renew. Mater..

